# Insights into phylogenetic relationships in *Pinus* inferred from a comparative analysis of complete chloroplast genomes

**DOI:** 10.1186/s12864-023-09439-6

**Published:** 2023-06-22

**Authors:** Qijing Xia, Hongbin Zhang, Dong Lv, Yousry A. El-Kassaby, Wei Li

**Affiliations:** 1 State Key Laboratory of Tree Genetics and Breeding, College of Biological Sciences and Technology, Beijing Forestry University, Beijing, 100083 China; 2 Gansu Province Academy of Qilian Water Resource Conservation Forests Research Institute, Zhangye, 734031 China; 3 Department of Forest and Conservation Sciences, Faculty of Forestry, University of British Columbia, Vancouver, Canada

**Keywords:** *Pinus*, Complete chloroplast genome, Comparative analysis, Phylogenetic relationships

## Abstract

**Background:**

*Pinus* is the largest genus of Pinaceae and the most primitive group of modern genera. Pines have become the focus of many molecular evolution studies because of their wide use and ecological significance. However, due to the lack of complete chloroplast genome data, the evolutionary relationship and classification of pines are still controversial. With the development of new generation sequencing technology, sequence data of pines are becoming abundant. Here, we systematically analyzed and summarized the chloroplast genomes of 33 published pine species.

**Results:**

Generally, pines chloroplast genome structure showed strong conservation and high similarity. The chloroplast genome length ranged from 114,082 to 121,530 bp with similar positions and arrangements of all genes, while the GC content ranged from 38.45 to 39.00%. Reverse repeats showed a shrinking evolutionary trend, with IRa/IRb length ranging from 267 to 495 bp. A total of 3,205 microsatellite sequences and 5,436 repeats were detected in the studied species chloroplasts. Additionally, two hypervariable regions were assessed, providing potential molecular markers for future phylogenetic studies and population genetics. Through the phylogenetic analysis of complete chloroplast genomes, we offered novel opinions on the genus traditional evolutionary theory and classification.

**Conclusion:**

We compared and analyzed the chloroplast genomes of 33 pine species, verified the traditional evolutionary theory and classification, and reclassified some controversial species classification. This study is helpful in analyzing the evolution, genetic structure, and the development of chloroplast DNA markers in *Pinus*.

**Supplementary Information:**

The online version contains supplementary material available at 10.1186/s12864-023-09439-6.

## Introduction

*Pinus* (Pinaceae) is the largest conifer genus among existing gymnosperms with more than 110 identified species. The genus natural distribution is mainly in the northern hemisphere, but it has been introduced and cultivated as a planation species all over the world [1]. As the most primitive group in modern genera of Pinaceae, *Pinus* has a long evolutionary history. Its fossil records can be traced back to 100 MYA [1–3], with a great potential for studying conifers evolutionary classification and species differentiation [3–6]. Pines are the main component of northern temperate forest and arid forest land, and are also important source of afforestation and industrial processing raw materials as well as their important ecological and economic values [7].

*Pinus* classification has always been a hot topic in phylogeny. Little et al. [8] proposed a classification system that divides *Pinus* into 3 Subgenera, 5 Sections, 15 Subsections and 94 species, and determined their basic classification framework. With scientific and technological advancements, *Pinus* classification system has gone through several revisions and improvements [1, 6, 9, 10]. Notably, Gernandt et al. [9] divided *Pinus* into 2 Subgenera (Subgenus: *Strobus* and *Pinus*), 4 Sections (Sections: *Trifoliae*, *Pinus*, *Parrya*, and *Quinquefoliae*) and 11 Subsections (Subsections: *Pinus*, *Pinaster*, *Contortae*, *Australes*, *Ponderosae*, *Balfourianae*, *Cembroides*, *Nelsoniae*, *Kremfianae*, *Gerardianae*, and *Strobus*) based on chloroplast gene sequence, nuclear DNA, and morphological evidence of 101 species. This classification system has been widely recognized [5, 11, 12]. However, the classification of individual species at the Subsection level has been controversial. Since *Pinus squamata* discovery, its classification efforts have been a hot issue. Li Xiangwang [13] discovered *P. squamata* and thought that it is close to *P. bungeana*. Price [14] pointed out that *P. squamata* may be a component of Subsection *Gerardianae*, or it may represent a separate Subsection. Li Xiangping et al. [15] incorporated *P. squamata* into Subsection *Balfourianae*. With wood anatomical data, Wang Changming et al. [16] supported the view that *P. squamata* is close to *P. bungeana*. In Gernandt et al. [9] traditional classification, *P. squamata* is also classified into the Subsection *Gerardianae* where *P. bungeana* and *P. gerardiana* are located. Although it is more likely that *P. squamata* belongs to Subsection *Gerardianae*, previous studies only relied on morphology and limited DNA data.

The chloroplast genome structure of terrestrial plants is stable [17] and has a large amount of genetic information, which can be used for phylogenetic inference and species classification [18]. In previous studies, chloroplast sequences have been extensively utilized as molecular markers in plant phylogeny research. However, due to the lack of complete chloroplast genome sequence data, many studies on chloroplast genome were limited to only few fragments, so the application of complete chloroplast genome sequence to phylogeny has not been widely applied [19–26]. The complete chloroplast genome sequence is much better than some fewer fragments in species phylogeny and classification determination [27–29]. With the development of new generation sequencing technology, phylogenetic analyses have ushered in a new era [30] and made it easier to obtain complete chloroplast genome sequences for many species. A large number of sequence data provide basic data for chloroplast genome structure study, gene composition, and also lay a foundation for plants phylogeny, classification, and species identification.

In this study, the complete chloroplast genomes of 33 published species of *Pinus* were characterized, and used to conduct genome comparative and phylogenetic analyses. We aimed to: (1) explore the size and structure differences of complete chloroplast genomes among the studied species; (2) identify highly variable regions in the studied chloroplast genomes; and (3) reconstruct pines phylogenetic relationship, and verify and supplement the traditional classification system.

## Results

### Characteristics of ***Pinus*** chloroplast (cp.) genomes

**Fig. 1:**
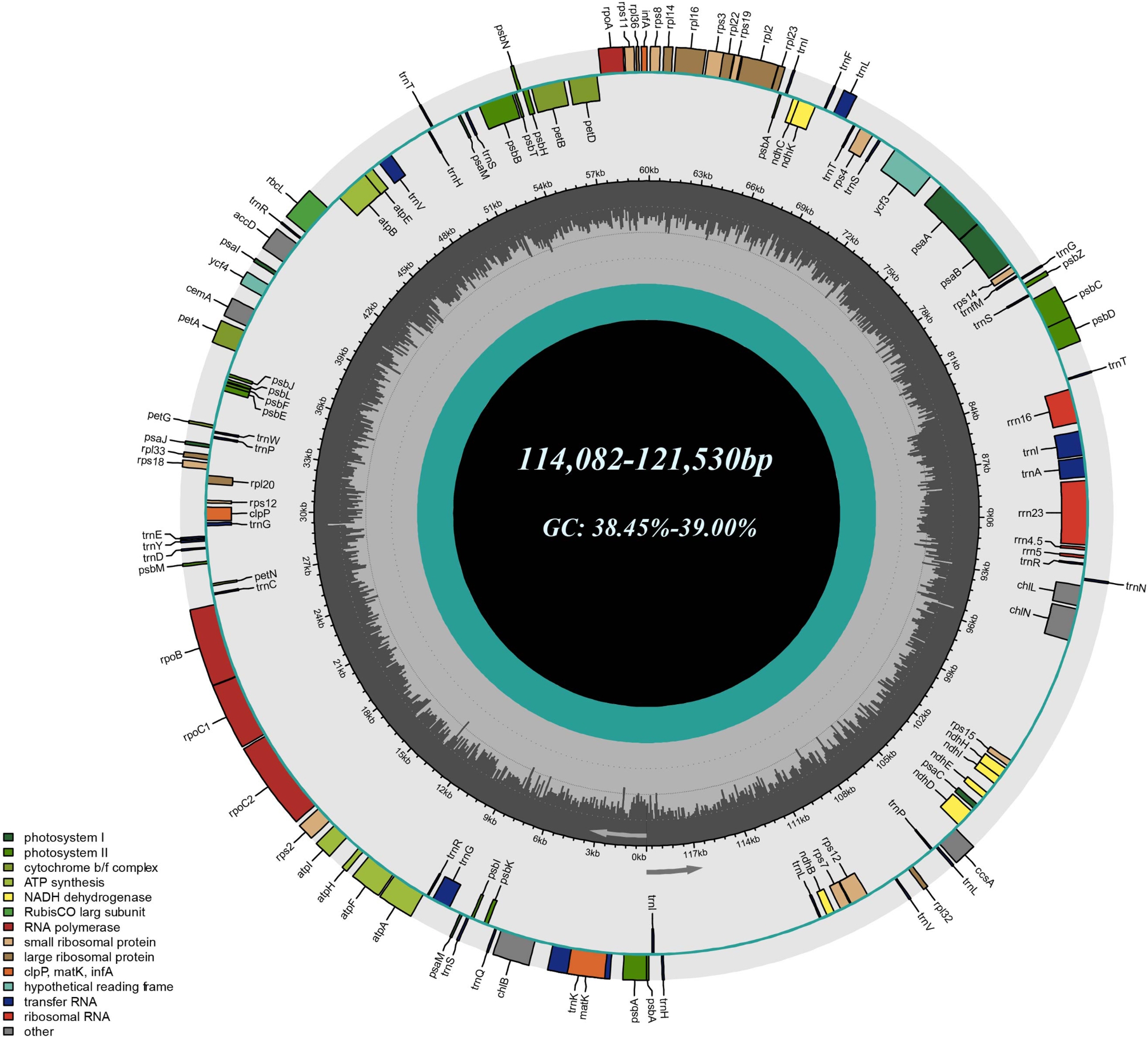
Gene cycle maps of 33 *Pinus* species. Color bars represent different functional groups. The dark and light gray columns in the inner circle correspond to the GC and AT contents, respectively

The cp. genomes of the 33 published pine species presented typical chloroplast genome structure, which consisted of a pair of inverted repeats (IRa/b) that divided into two single-copy regions: large single-copy (LSC) and small single-copy (SSC) regions (Fig. 1). Chloroplast genomes sequence similarity among 33 species was more than 95%. There was no significant difference in the size, gene, and genome structure among the studied chloroplast genomes. The genomes’ quadripartite structure was not obvious, which was mainly manifested by the reduction of the IR regions. The chloroplast genome length ranged from 114,082 to 121,530 bp, LSC region of which ranged from 62,747 to 66,364 bp, SSC region ranged from 49,112 to 54,288 bp, and IR regions ranged from 267 to 495 bp. The species with the largest chloroplast genome length was *P. taeda*, and the smallest was *P. pinceana.* The chloroplast genome size of *Pinus* was lower than that of most other seed plants, which may be related to the reduction of IR regions during evolution. Total GC content was 38.45-39.00%, with no significant difference among the 33 species (Table 1). The GC content of the genome was an important indicator to judge the genetic relationship between species, which further showed that the chloroplast genomes of the 33 pine species were highly similar.

**Table 1:** Summary of *Pinus* chloroplast genome features

Species	Accession number	Genome size(bp)					GC% AT%	
		LSC	SSC	IRA	IRB	Total		
***Pinus aristata***	NC_039809.1	65,192	52,606	312	312	118,422	38.62	61.38
***Pinus armandii***	NC_029847.1	64,548	51,767	475	475	117,265	38.79	61.21
***Pinus bungeana***	NC_028421.1	65,373	51,538	475	475	117,861	38.83	61.17
***Pinus contorta***	MH612863.1	65,836	54,131	267	267	120,501	39.00	61.00
***Pinus crassicorticea***	NC_041150.1	65,737	53,216	388	388	119,729	38.55	61.45
***Pinus densiflora***	NC_042394.1	65,654	53,231	495	495	119,875	38.49	61.51
***Pinus elliottii***	NC_042788.1	65,600	53,308	484	484	119,876	38.46	61.54
***Pinus gerardiana***	NC_011154.4	65,131	51,771	358	358	117,618	38.90	61.10
***Pinus greggii***	NC_035947.1	65,536	53,995	485	485	120,501	38.45	61.55
***Pinus jaliscana***	NC_035948.1	65,553	54,192	485	485	120,715	38.46	61.54
***Pinus koraiensis***	NC_004677.2	64,523	51,717	475	475	117,190	38.80	61.20
***Pinus krempfii***	NC_011155.4	65,036	51,257	348	348	116,989	38.91	61.09
***Pinus lambertiana***	NC_011156.4	64,578	51,715	473	473	117,239	38.79	61.21
***Pinus massoniana***	NC_021439.1	65,557	53,212	485	485	119,739	38.55	61.45
***Pinus monophylla***	NC_011158.4	64,752	50,811	458	458	116,479	38.73	61.27
***Pinus morrisonicola***	NC_039616.1	64,104	51,770	381	381	116,636	38.75	61.25
***Pinus nelsonii***	NC_011159.4	64,935	50,991	454	454	116,834	38.89	61.11
***Pinus oocarpa***	NC_035949.1	65,485	54,141	485	485	120,596	38.47	61.53
***Pinus parviflora***	NC_039615.1	66,364	53,410	475	475	120,724	38.58	61.42
***Pinus pinceana***	NC_039587.1	64,346	49,112	312	312	114,082	38.81	61.19
***Pinus pinea***	NC_039585.1	65,357	53,634	490	490	119,971	38.45	61.55
***Pinus pumila***	NC_041108.1	64,606	51,844	475	475	117,400	38.80	61.20
***Pinus sibirica***	NC_028552.2	63,908	51,781	473	473	116,635	38.72	61.28
***Pinus squamata***	NC_039614.1	64,706	51,825	398	398	117,327	38.73	61.27
***Pinus strobus***	NC_026302.1	62,747	51,885	472	472	115,576	38.77	61.23
***Pinus sylvestris***	NC_035069.1	65,559	53,209	495	495	119,758	38.50	61.50
***Pinus tabuliformis***	NC_028531.1	65,618	53,038	495	495	119,646	38.53	61.47
***Pinus taeda***	NC_021440.1	66,272	54,288	485	485	121,530	38.50	61.50
***Pinus taiwanensis***	NC_027415.1	65,670	53,081	495	495	119,741	38.51	61.49
***Pinus teocote***	NC_039586.1	65,516	53,910	485	485	120,396	38.46	61.54
***Pinus thunbergii***	NC_001631.1	65,696	53,021	495	495	119,707	38.50	61.50
***Pinus wangii***	NC_039613.1	65,600	51,521	476	476	118,073	38.70	61.30
***Pinus yunnanensis***	NC_043856.1	65,619	53,098	495	495	119,707	38.52	61.48

All chloroplast genomes contained a total of 108 genes, including 72 protein-coding (PCGs), 32 tRNA, and four rRNA genes. Only the *trnI-GAU* gene and part of *psbA* gene were distributed in the IR region. All genes had the same location and arrangement across the different chloroplast genomes (Table 2). Among the above annotated genes, 14 genes contained introns, including 8 PCGs (*atpF*, *petB*, *petD*, *rpl2*, *rps12*, *rpl16*, *rpoC1*, and *ycf3*) and 6 tRNA (*trnV-UAC*, *trnL-UAA*, *trnK-UUU*, *trnI-GAU*, *trnG-UCC* and *trnA-UGC*) genes. Among them, *rps12* and *ycf3* contained two introns, the remaining 12 contained one intron; *matK* was located on the intron of *trnK-UUU*; *trnH-GUG*, *trnI-CAU*, *trnS-GCU*, *trnT-GGU*, *psbA* and *psaM* had two gene copies in the genome. In addition, as in angiosperms, *rps12* was also trans-spliced during transcription in *Pinus*.

**Table 2:** List of genes annotated in the chloroplast genome of *Pinus* species

Function	Genes
Ribosomal RNAs	*rrn4.5, rrn5, rrn16, rrn23*
Transfer RNAs	trnA-UGC*, trnC-GCA, trnD-GUC, trnE-UUC, trnF-GAA, trnG-GCC, trnG-UCC*, ***trnH-GUG***, ***trnI-CAU***, trnI-GAU*, trnK-UUU*, trnL-CAA, trnL-UAA*, trnL-UAG, trnM-CAU, trnfM-CAU, trnN-GUU, trnP-UGG, trnP-GGG, trnQ-UUG, trnR-ACG, trnR-CCG, trnR-UCU, ***trnS-GCU***, *trnS-GGA, trnS-UGA*, ***trnT-GGU***, trnT-UGU, trnV-GAC, trnV-UAC*, trnW-CCA, trnY-GUA
RNA polymerase	rpoA, rpoB, rpoC1*, rpoC2
Maturase	*matK*
Ribosomal proteins (SSU)	rps2, rps3, rps4, rps7, rps8, rps11, rps12**^, *T*^, *rps14, rps15, rps18, rps19*
Ribosomal proteins (LSU)	rpl2*, rpl14, rpl16*, rpl20, rpl22, rpl23, rpl32, rpl33, rpl36
ATP synthase	atpA, atpB, atpE, atpF*, atpH, atpI
Photosystem I	*psaA, psaB, psaJ*, ***psaM***, *psaC, psaI*
Photosystem II	*psbI, psbJ, psbH, psbT, psbN, psbM, psbK, psbD*, ***psbA***, *psbL, psbC, psbE, psbF, psbB, psbZ*
RubisCO large subunit	*rbcL*
Cytochrome b/f complex	petL, petA, petB*, petG, petN, petD*
Chlorophyll biosynthesis	*chlB, chlL, chlN*
*Protease*	*clpP*
Acetyl-CoA carboxylase	*accD*
Inner membrane protein	*cemA*
Cytochrome c biogenesis	*ccsA*
Translation initiation factor	*infA*
Hypothetical chloroplast reading frames(ycf)	ycf1, ycf2, ycf3**, ycf4, ycf12

*Genes containing one intron; ** genes containing two introns; ^T^ trans-splicing of the related gene. Genes in boldface type have two gene copies

The number and sequence of rRNA genes were the same as those of “typical” seed plant plastids such as *Nicotiana*, and they were all arranged in the order of 16 S, 23 S, 4.5 and 5 S rRNA [31]. However, there were some differences in the content of other genes between *Pinus* and angiosperms. Angiosperms lost *trnP-GGG* and three *chl* genes (*chlB*, *chlL*, *chlN*) during evolution [32]. The gene *rpl23* deletion had been reported in the plastids of angiosperms *Spinacia* [33, 34] and *Trachelium* [32]. The gene *rps16* had experienced many independent losses in land plants [32, 35, 36]. Similarly, the chloroplasts of *Pinus* also lacked *rps16*. In addition, unlike *Pinus*, in many prokaryote and eukaryote lineages, the gene *accD* had been lost independently [37].

### Highly variable regions in the ***Pinus*** chloroplast genomes

**Fig. 2:**
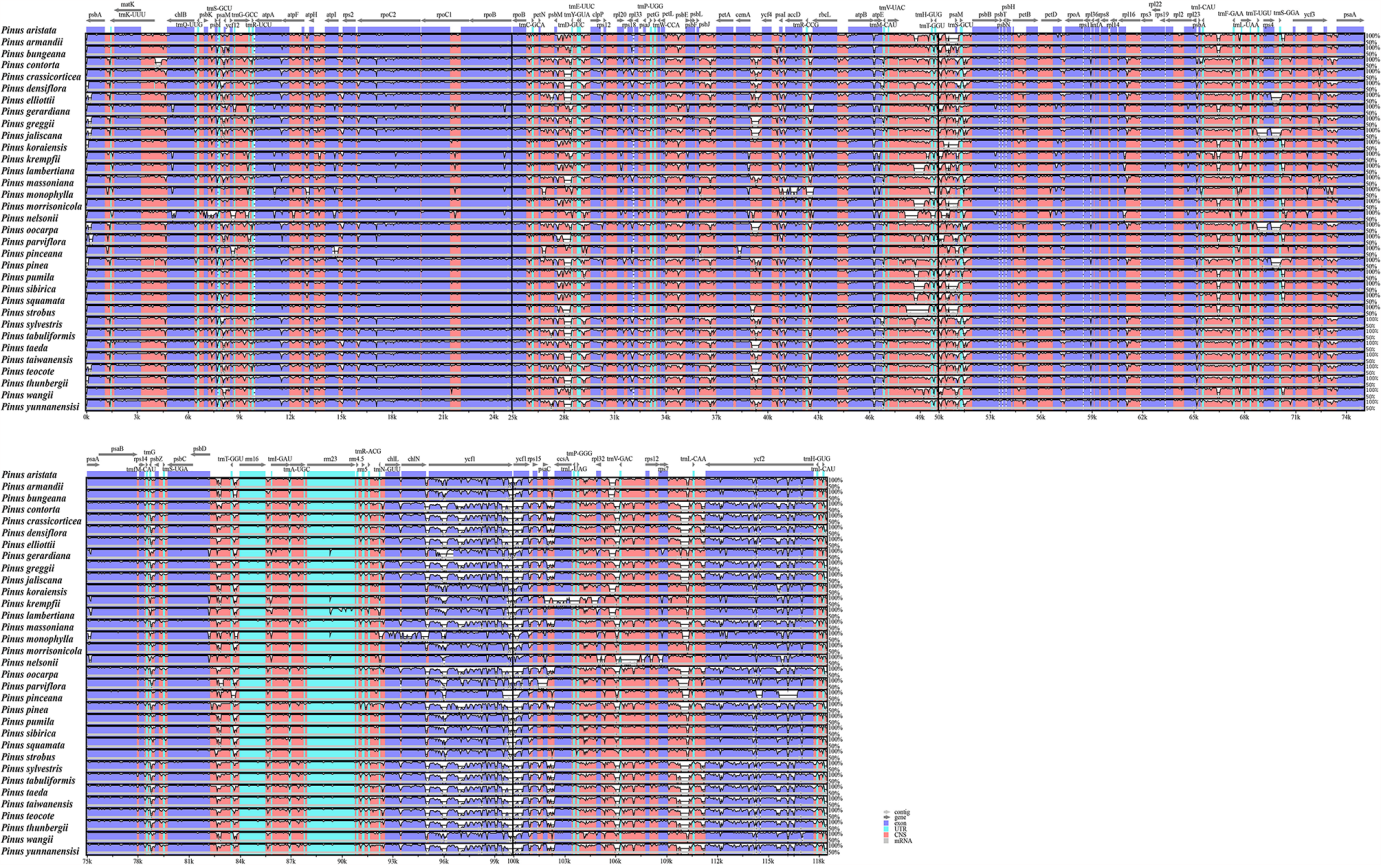
Visualization of genome alignment of the complete chloroplast genome of 33 *Pinus*. The cp. genome of *P. armandii* was used as reference. X-axis indicates the sequence coordinates in the whole cp. genome. Y-axis represents the similarity of the aligned regions, indicating percent identity to the reference genome (50–100%)

**Fig. 3:**
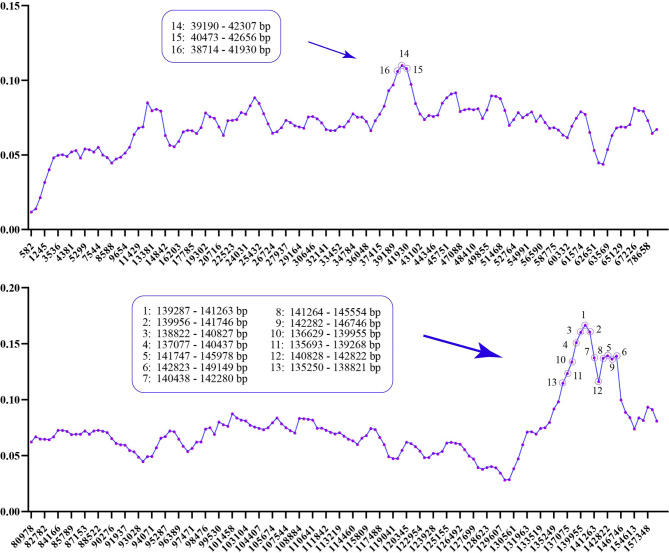
Sliding-window analysis showing the nucleotide diversity (Pi) values of the aligned *Pinus* chloroplast genomes. The dimension areas are the 16 areas with the highest Pi value

The comparative visualization of the complete chloroplast genomes of the 33 species clearly showed sequences differences. As a whole, all genomes were relatively conservative and the variation of most coding genes and all rRNAs was relatively small. The regions with obvious gap were mostly concentrated in non-coding regions, among which *psbM-trnD*, *cemA-ycf4*, *trnV-trnH*, *trnT-psbM*, *trnT-rps4-trnS*, *psbD-trnT-rrn16*, *psaC-ccsA*, *rpl32-trnV* and *rps7-trnL* were the most significant; and in the coding regions, *atpE*, *ycf1* and *ycf2* were the most significant (Fig. 2). In order to further analyze the differences in the studied 33 *Pinus* chloroplast genomes, we identified highly variable regions by calculating the nucleotide diversity (Pi). Two highly variable regions *psbM-trnD-trnY-trnE-clpP-rps12* and *chlN-ycf1* were obtained by screening the 16 regions with the highest Pi value (0.10616–0.16672) (Fig. 3; Table S1). Chloroplast genome rearrangement analysis results showed that rearrangement events of genome were not obvious (Fig. S1).

### The chloroplast genomes of ***Pinus*** have a contracted IR region

**Fig. 4:**
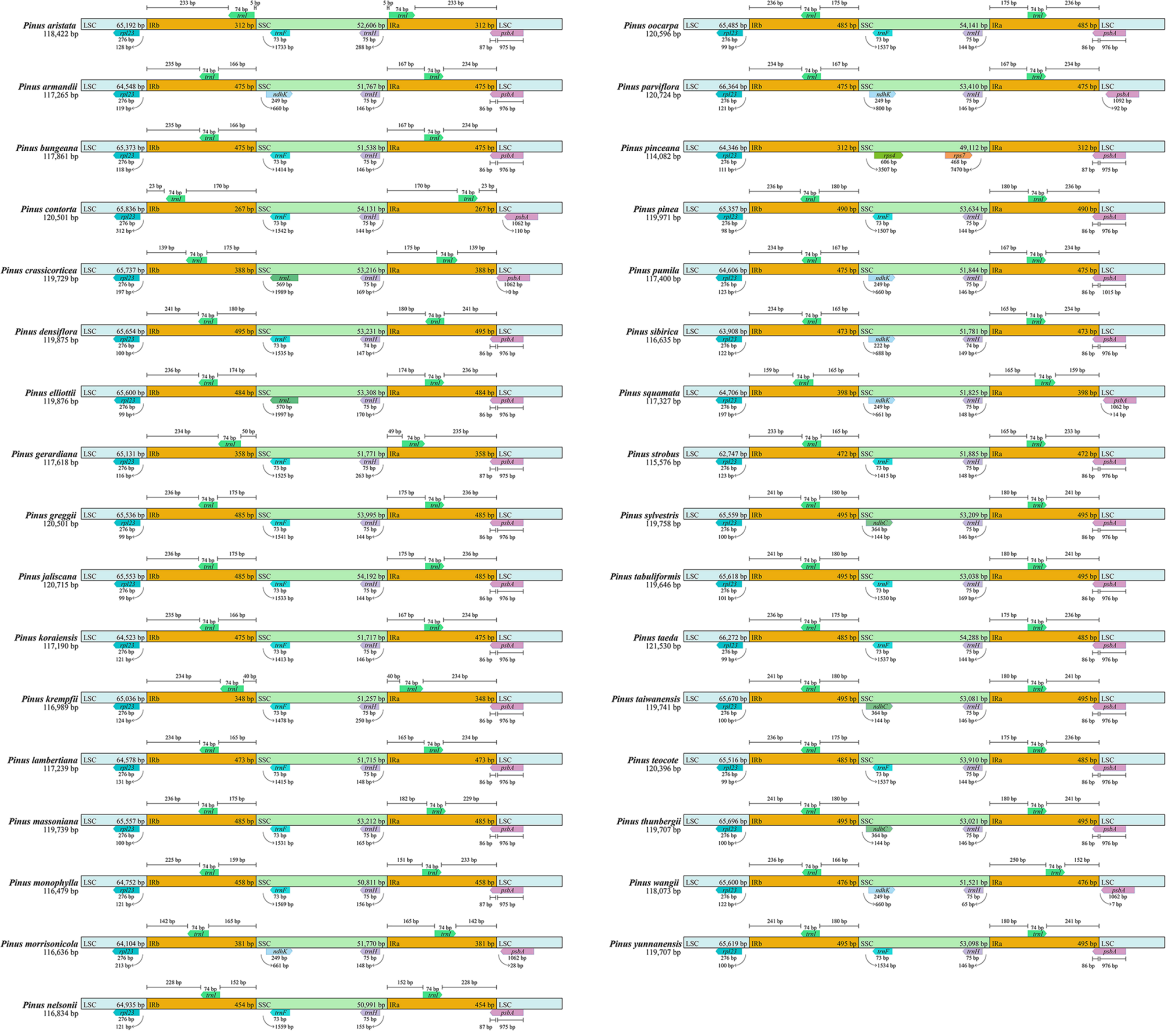
Comparison of the Large Single-Copy (LSC), Small Single-Copy (SSC), and inverted repeat (IR) boundary regions across the 33 *Pinus* chloroplast genomes

The single copy and inverted repeat boundary maps of the 33 species showed that, similar to most terrestrial plants, the cpDNA genome could be divided into four parts, including LSC, SSC, and two IR regions that separated them. However, the difference was that the IR regions of *Pinus* was not complete as they lost a large number of reverse repeat copies during their evolution. The IR regions had shrunk significantly, with a size of only 267–495 bp. Only *trnI* gene and part of *psbA* gene were retained in IRa region, and only *trnI* was retained in IRb region. The size range of LSC was 62,747 − 66,364 bp, and the size range of SSC was 49,112 − 54,288 bp, yet the size difference between the two regions was not obvious. With the exception of 6 species (*P. contorta*, *P. crassicorticea*, *P. morrisonicola*, *P. parviflora, P. squamata, P. wangii*), the IRa/LSC junction in the chloroplast genomes of the other 28 species was located in *psbA*, and the range extending to the IRa region was 86–87 bp (Fig. 4).

### SSRs and long repeats analysis

**Fig. 5:**
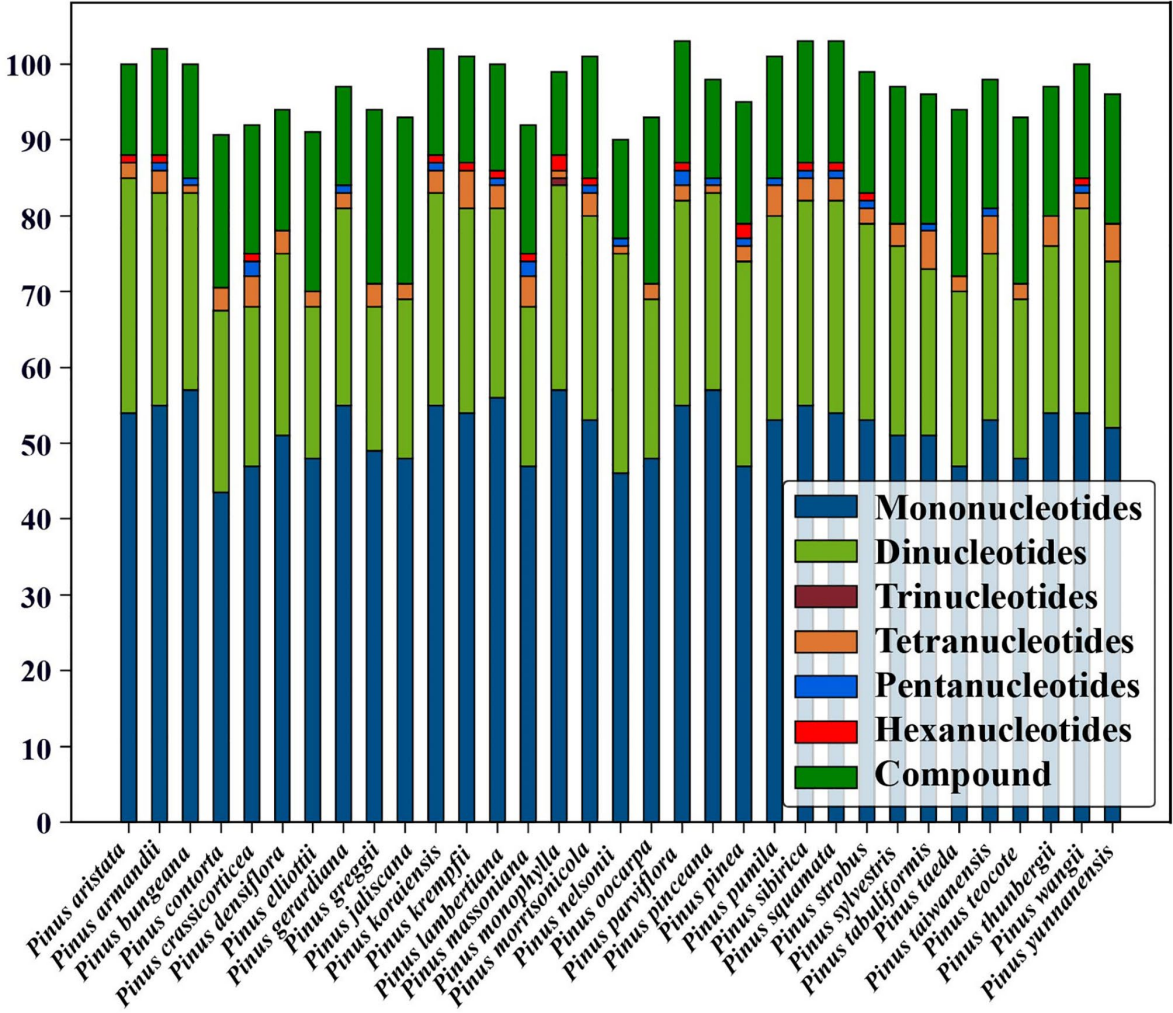
Numbers and types of simple sequence repeats (SSR) in the 33 *Pinus* chloroplast genomes

A total of 3,205 simple sequence repeats (SSRs) with a length ranging from 8 to 230 bp were detected in the studied 33 species. Among them, there were 1,708 mononucleotide repeats with the highest frequency, mainly A or T single nucleotide, with obvious base preference. The rest were dinucleotide (817), compound (548), tetranucleotide (92), pentanucleotide (22), hexanucleotide (17), and trinucleotide repeats (1). The number of trinucleotide repeats was the least, and it appeared only once in *P. monophylla*. Only 4 types of SSRs were detected in 10 species, all of which lacked trinucleotide, pentanucleotide, and hexanucleotide repeats. The comparison results among the 33 species showed that the largest number of SSRs (103) appeared in *P. parviflora*, *P. sibirica*, and *P. squamata*, and the smallest number (90) appeared in *P. nelsonii* (Fig. 5; Table S2).

**Fig. 6:**
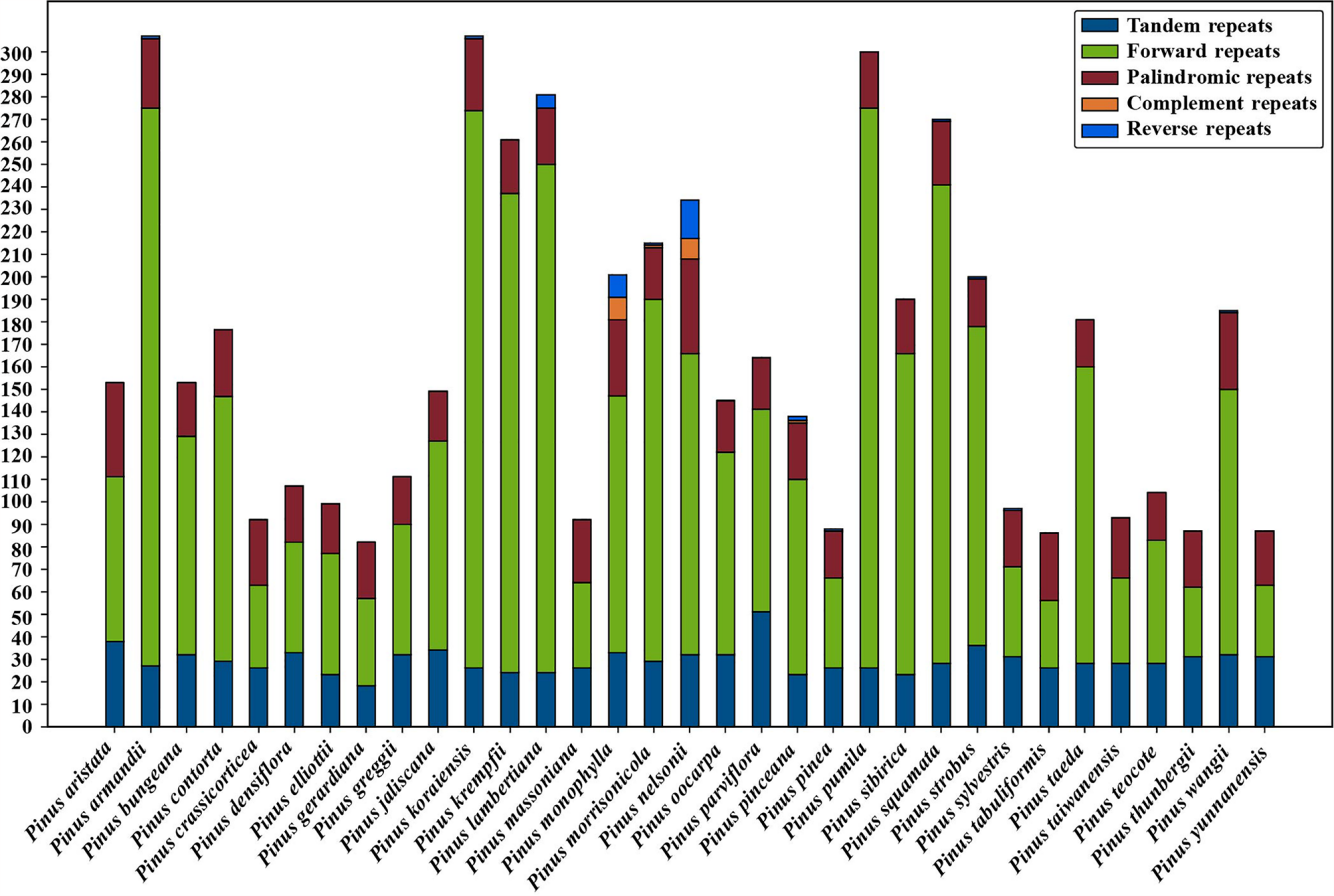
Analyses of repeated sequences in complete chloroplast genomes of 33 *Pinus* species

A total of 5,436 long repeats were detected across the 33 species, including tandem (965), forward (3531), palindromic (876), complement (21), and reverse (43) repeats. Among these sequences, forward repeats were the most abundant. The species comparison results showed that *P. armandii* and *P. koraiensis* contained the largest number of repeat sequences (307), and *P. gerardiana* was the least (82). The difference of forward repeats among species was the most obvious, and the difference between the species *(P. pumila*) with the largest number and the species (*P. tabuliformis*) with the smallest number was 219. The number of tandem repeats and palindromic repeats was similar, the species with the largest number of tandem repeats was *P. parviflora* (51), and the species with the largest number of palindromic repeats were *P. aristata* (42) and *P. nelsonii* (42). The number of complement repeats was the least, and only appears in 4 species (*P. monophylla*, *P. morrisonicola*, *P. nelsonii*, *P. pinceana*). There were no reverse repeats detected in 21 species (Fig. 6; Table S3).

### Revisiting the phylogenetic relationships with complete chloroplast genomes

**Fig. 7:**
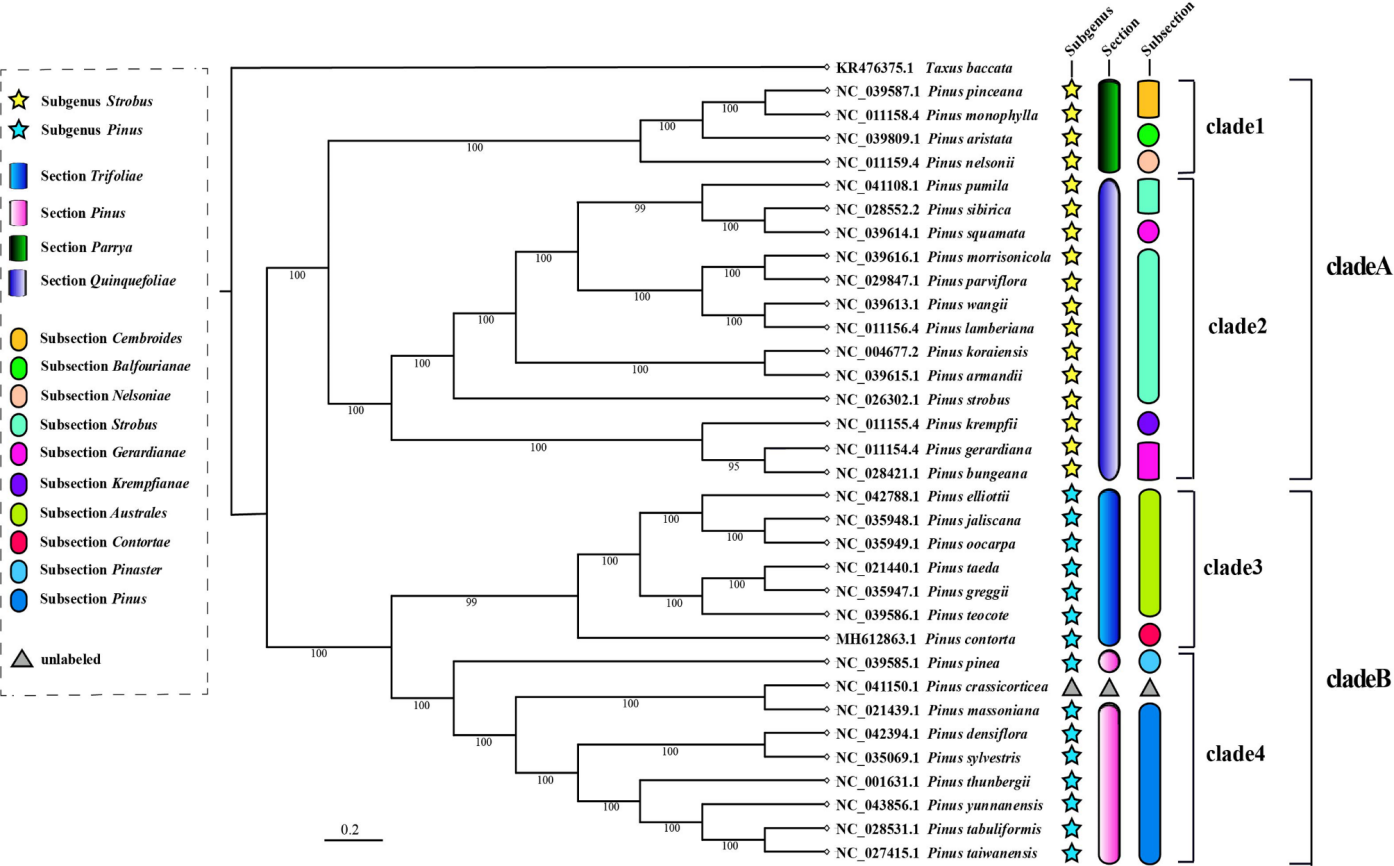
Maximum-likelihood phylogenetic tree based on complete chloroplast genome sequences of 33 *Pinus* species. *Taxus baccata* was used as outgroup

The complete chloroplast genomes of the 33 species were analyzed by maximum likelihood (ML) method. Gernandt et al. [9] proposed a traditional classification system through chloroplast gene sequences, based on which we annotated the phylogenetic results. The 33 studied species cover 2 Subgenera, 4 Sections, and 10 Subsections of the traditional classification system. The phylogenetic tree showed that the 33 species were divided into 2 large branches and 4 small branches, which were consistent with the traditional classification system. This result strongly supported the feasibility of Subgenus and Section in the traditional classification. However, there were still some issues in the Subsections division. Gernandt et al. [9] classified *P. squamata* as Subsection *Gerardianae*, but our phylogenetic analysis results were not supportive. *P. squamata* and species in the Subsection *Strobus* were clustered into one branch, and were closest to *P. sibirica* in the Subsection *Strobus*. Therefore, it could be considered to be included in Subsection *Strobus*. In addition, *P. crassicorticea*, which had never been mentioned in the traditional classification system, was classified as Subgenus *Pinus*, Section *Pinus*, Subsection *Pinus* according to its phylogenetic position (Fig. 7).

## Discussion

### IR regions reduction resulted in variable cpDNA sizes in ***Pinus***

Chloroplast genomes of most terrestrial plants were composed of double stranded closed circular DNA molecules with conservative structure and typical quadripartite structure, including a LSC, a SSC, and two IR regions separated by LSC and SSC regions [17]. Although the chloroplast genomes of most gymnosperms, such as cycads, Ginkgo and Gnetophytes, had the typical quadripartite structure of seed plants [38–41], they had changed in the chloroplast genomes of Pinaceae and Cupressophytes. In previous studies, it was proposed that the IR was highly simplified in Pinaceae, but completely lost in Cupressophytes, and Pinaceae and Cupressophytes lost different IR copies, Pinaceae lost IRb, and Cupressophytes lost IRa [42, 43]. *P. thunbergii* in *Pinus* also proved that each IR region was shortened to 495 bp [44]. Our results were similar to the previous conclusions, the quadripartite structure of the studied 33 pine species was not obvious, and the size of each IR region is only 267–495 bp, showing a decreasing trend. However, IRa and IRb did not differ in size, and also did not reflect the IRb loss (Table 1). In addition, the results showed that there was no significant difference in the size of LSC and SSC regions, and there was a possibility that part of IR region could be translocated into SSC region. The chloroplast genome of seed plants usually contains 101–118 different genes [45], and the genome size ranges from 120 to 160 kb [46]. The studied 33 pine species contained 108 different genes, and the size of chloroplast genome ranged from 114,082 to 121,530 bp (Tables 1 and 2). It can be seen that the reduction of IR region resulted in the size of chloroplast genome, and the types of genes in *Pinus* are lower than those in other seed plants. Although the chloroplast genomes of Pinaceae and Cupressophytes do not contain typical IR, they still evolve specific IR related to chloroplast genome rearrangement. The chloroplast genomes of some conifers have shown very low collinearity [43, 47]. Strauss et al. [48] also speculated that in Pinaceae cpDNA, rearrangement may occur after IR reduction. However, genome synteny (Fig. S1) of *Pinus* chloroplast genomes revealed no obvious gene rearrangement events. This may be related to the strong conservation and high similarity of pines chloroplast genome structure.

### Significance of chloroplast markers in population genetics

The existence and nature of repeat sequences had been proven to be of great significance for evolution and population genetics studies [49, 50]. A total of 7 types of SSRs were detected in the 33 pine species, of which 1,078 were mononucleotide repeats, mainly A or T single nucleotide, with base preference (Fig. 5; Table S2). The A/T base preference of pines chloroplast genomes was the same as that of many seed plants, SSRs were usually composed of polyA or polyT repeat sequences [51–54]. Recently, genomic SSRs markers have been widely used in *Pinus* [55–57]. However, compared with genomic SSRs, chloroplast SSRs markers were abundant in number, high in polymorphism and rich in species variability [58]. The newly discovered SSRs in this study will contribute to future studies on *Pinus* genetic diversity and phylogeography. Pines are rich in long repeats, a total of 5,436 repeats were detected in the studied 33 species, of which forward repeats had the highest frequency (Fig. 6; Table S3). All repeats detected in this study, together with the above SSRs, had laid a foundation for the development of population genetic markers [59].

We screened 16 regions with the highest Pi values among the studied 33 pines, the regions they represent were *psbM-trnD-trnY-trnE-clpP-rps12* and *chlN-ycf1* (Fig. 3; Table S1). These two highly variable regions will provide potential molecular markers for population genetics studies. In gymnosperms, chloroplasts were generally inherited by paternity [60, 61]. Therefore, the highly variable regions detected in the present study can provide information for the development of specific DNA bar codes of *Pinus*, and then serve as an effective means to identify male pines parents.

### Phylogenetic analysis of complete chloroplast genome reconstruction

Chloroplast genome was characterized by abundant gene capacity, conservative structure, low evolution rate, and high copy number. It had always been the main object of phylogenetic and molecular evolution research [45, 62]. Studies on the phylogeny of chloroplast genome initially relied on single gene sequences [63, 64], but single gene sequences contained less information, resulting in low support rates for many branches [30, 65, 66]. With the accumulation of data, the resolution and support rate of multi gene joint sequence reconstruction phylogenetic analysis had been significantly improved [67–69], and had been widely used [18, 20]. Among them, Gernandt et al. [9] conducted phylogenetic analysis based on chloroplast *matK* and *rbcL* sequences of 101 species of pines and constructed the classification system of *Pinus*. However, with the accumulation of complete genome data of *Pinus* chloroplasts, it was necessary to verify the traditional classification system. In this study, we reconstructed the phylogenetic relationships of the complete cp. genomes of the 33 pine species. Except for *P. squamata*, the classification of other species was consistent with the traditional results. Different from previous research results [9, 13–16], this study supported *P. squamata* to join Subsection *Strobus* (Fig. 7). Similarly, *P. nelsonii*, *P. krempfii*, and *P. contorta* also had the problem of unclear classification in previous studies [70], and the present study also gave reference which supported *P. nelsonii* joining Section *Parrya*, *P. krempfii* joining Section *Quinquefoliae* and *P. contorta* joining Section *Trifoliae*. This work is helpful to further understanding the evolution of chloroplasts in *Pinus* and will promote the research progress of pines phylogeny and taxonomy.

## Conclusions

We conducted comparative and phylogenetic analyses of the complete chloroplast genomes of 33 pine species. *Pinus* chloroplast genomes structure was conservative, sequence similarity was high, and the IR region showed a decreasing trend. The discovery of two highly variable regions provided reference information for the development of *Pinus* chloroplast DNA bar code for future use. We reconstructed the phylogenetic relationship among the 33 pine species using the complete chloroplast genomes, which provided better resolution than that from traditional chloroplast DNA sequences. According to the phylogenetic results, we verified the traditional classification system and revised the position of *P. squamata*. With the increasing abundance of chloroplast genome information in *Pinus*, the systematic analysis and summary will enhance our understanding of *Pinus* evolutionary history, phylogeny, and taxonomy.

## Materials and methods

### Data collection and processing

The chloroplast genome sequences of 33 published pine species were downloaded from NCBI, including *P. taristata*, *P. armandii*, *P. bungeana*, *P. contorta*, *P. crassicorticea*, *P. densiflora*, *P. elliottii*, *P. gerardiana*, *P. greggii*, *P. jaliscana*, *P. koraiensis*, *P. krempfii*, *P. lambertiana*, *P. massoniana*, *P. monophylla*, *and P. morrisonicola*, *P. nelsonii*, *P. oocarpa*, *P. parviflora*, *P. pinceana*, *P. pinea*, *P. pumila*, *P. sibirica*, *P. squamata*, *P. strobus*, *P. sylvestris*, *P. tabuliformis*, *P. taeda*, *P. taiwanensis*, *P. teocote*, *P. thunbergii*, *P. wangii*, and *P. yunnanensis*. The sequences of 33 complete chloroplast genomes were aligned using MAFFT v7.0 [71] and then manually checked and modified for subsequent analysis.

### Comparative genomic analysis

mVISTA v.7 program [72] was used for multiple sequence alignment analysis, and the sequences were processed by CPGAVAS2 (http://www.herbalgenomics.org/cpgavas). Considering the chloroplast genome of *P. armandii* as a reference, the differences of the whole chloroplast genome of the 33 pine species were compared under the Shuffle-LAGAN model. Nucleotide diversity was used as a parameter to identify the cp. genome highly variable region. Here, we used DnaSP v.6.1 [73] software to estimate nucleotide diversity, the step length and window length were set to 200 and 800 bp, respectively, then used GraphPad-prism v.9.0 (https://www.graphpad.com/scientific-software/prism) to visualize the data. Chloroplast genome rearrangement analysis was performed using the default settings of the Mauve v.2.3 [74] plug-in in Geneious v.11.0 [75].

### Detection of long repeat sequences and simple sequence repeats

The online REPuter (https://bibiserv.cebitec.uni-bielefeld.de/reputer) [76] was used to identify long repeats (tandem, forward, reverse, palindromic, and complement repeats). The minimum repetition size was limited to no less than 30 basis points, the Hamming distance value was 3, and other settings remained at the default value. The SSRs of the chloroplast genomes of the 33 pine species were identified by microsatellite marker identification tool (MISA) (https://webblast.ipk-gatersleben.de/misa), the minimum number of repeats was used to identify mononucleotides, dinucleotides, trinucleotides, tetranucleotides, pentanucleotides, and hexanucleotides were 8, 4, 4, 3, 3 and 3, respectively; the sequence length between two SSRs was no more than 100 bp, and it was registered as a compound [77].

### Phylogenetic analysis

In order to determine the phylogenetic location of the 33 pine species, we used the complete chloroplast genome sequences for phylogenetic analysis with *Taxus* as an outgroup. The complete chloroplast genome sequences were downloaded from NCBI. MAFFT v7.0 [71] was used for sequence alignment, and ModelFinder [78] was used to find the most suitable alternative models TVM + F + R2 for the complete chloroplast genome sequences. Phylogeny was constructed by ML analysis, and ML analysis was performed by IQ-tree v1.6 [79] with 1000 bootstrap repeats. Using Figtree v1.4 (https://github.com/rambaut/Figtree) edit the two phylogenetic trees.

## Electronic supplementary material

Below is the link to the electronic supplementary material.

Supplementary Material 1

Supplementary Material 2

Supplementary Material 3

Supplementary Material 4

## Data Availability

All data supporting the findings of this study are available within the paper and within its supplementary materials published online. All data used in the study were collected in the public database (https://www.ncbi.nlm.nih.gov/). Accession numbers of 33 species are as follow: *P. aristate*, NC_039809.1; *P. armandii*, NC_029847.1; *P. bungeana*, NC_028421.1; *P. contorta*, MH612863.1; *P. crassicorticea*, NC_041150.1; *P. densiflora*, NC_042394.1; *P. elliottii*, NC_042788.1; *P. gerardiana*, NC_011154.4; *P. greggii*, NC_035947.1; *P. jaliscana*, NC_035948.1; *P. koraiensis*, NC_004677.2; *P. krempfii*, NC_011155.4; *P. lambertiana*, NC_011156.4; *P. massoniana*, NC_021439.1; *P. monophyla*, NC_011158.4; *P. morrisonicola*, NC_039616.1; *P. nelsonii*, NC_011159.4; *P. oocarpa*, NC_035949.1; *P. parviflora*, NC_039615.1; *P. pinceana*, NC_039587.1; *P. pinea*, NC_039585.1; *P. pumila*, NC_041108.1; *P. sibirica*, NC_028552.2; *P. squamata*, NC_039614.1; *P. strobus*, NC_026302.1; *P. sylvestris*, NC_035069.1; *P. tabuliformis*, NC_028531.1; *P. taeda*, NC_021440.1; *P. taiwanensis*, NC_027415.1; *P. teocote*, NC_039586.1; *P. thunbergii*, NC_001631.1; *P. wangii*, NC_039613.1; *P. yunnanensis*, NC_043856.1.
