# Supplementary material for: Insights into phylogenetic relationships in Pinus inferred from a comparative analysis of complete chloroplast genomes
Source: BMC Genomics. 2023 Jun 22;24:346. doi: 10.1186/s12864-023-09439-6 (PMC10286357; doi:10.1186/s12864-023-09439-6)
Supplement: Supplementary file 4 — Supplementary Material 4 [file 12864_2023_9439_MOESM4_ESM.docx]

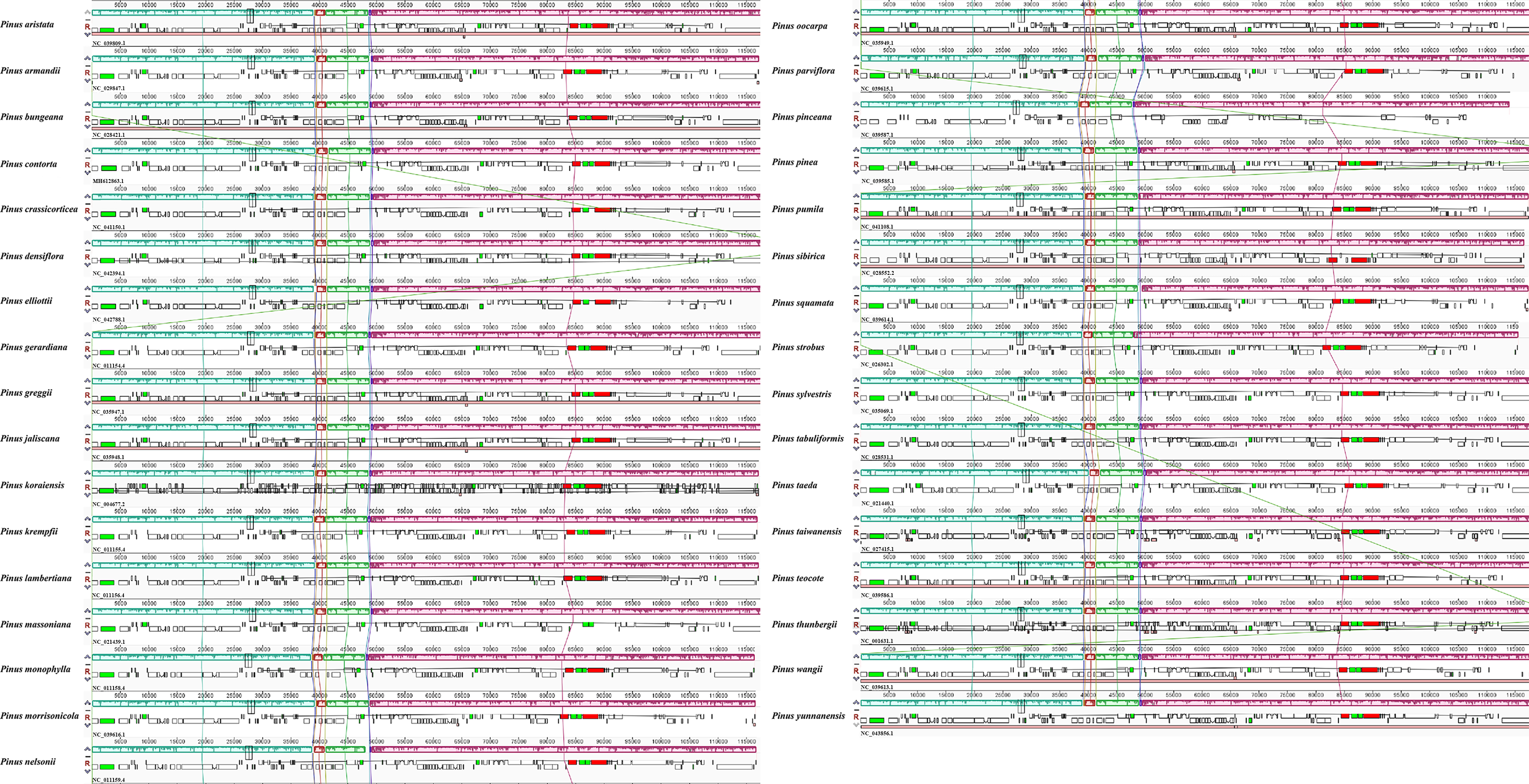


**Fig.S1** Genomic rearrangement of the 33 *Pinus* chloroplast genomes, with *P. armandii* set as a reference genome.
